# Defining digital surgery for the future

**DOI:** 10.1038/s41746-022-00706-6

**Published:** 2022-10-19

**Authors:** Marium M. Raza, Kaushik P. Venkatesh, James A. Diao, Joseph C. Kvedar

**Affiliations:** Harvard Medical School, Boston, MA USA

**Keywords:** Health policy, Technology

## Abstract

Innovations in robotics, virtual and augmented reality, and artificial intelligence are being rapidly adopted as tools of “digital surgery”. Despite its quickly emerging role, digital surgery is not well understood. A recent study defines the term itself, and then specifies ethical issues specific to the field. These include privacy and public trust, consent, and litigation.

Innovations in medical technology have transformed nearly every part of the healthcare landscape. In the fields of surgery, robotics, and virtual reality, artificial intelligence (AI) is being rapidly adopted. Surgical preplanning has been enhanced through computer-generated models and intraoperational navigational aids, especially in orthopedics, neurosurgery, and maxillofacial surgery. For example, AI and deep learning are being used for real-time identification of normal structures and malignant tissues^1^. The first commercial AI system for endoscopy, “GI Genius”, was approved by the US Food and Drug Administration (FDA) last year^2^. Additionally, automated analysis of surgical video and outcomes can provide a way to appraise and maintain standards, to make surgeries safer and more efficient.

Despite its quickly emerging role, the term “digital surgery” is not clearly defined or standardized. The careful definition is especially important given the unique challenges posed by surgical applications. Lam et al. use the Delphi technique to define digital surgery, characterize ethical and data governance issues, and identify barriers to its continued advancement^3^.

Originally developed to forecast military implications of new technology, the Delphi technique is a structured method for reaching consensus among experts. In the last few decades, it has been used to generate collective intelligence amid limited research or conflicting evidence^4^. Lam et al. recruited a panel of 38 experts, including surgeons interested in digital technology, academics, and healthcare industry professionals. The Delphi technique consists of four rounds. Round one asked experts and members of the public to ideate issues around themes they observed in academic literature or their clinical experiences. In the next two rounds, experts voted on the relative importance of the identified issues. In the final round, experts discussed and voted on consensus statements from previous rounds.

Through this procedure, all 38 experts reached consensus on the definition of digital surgery as “the use of technology for the enhancement of preoperative planning, surgical performance, therapeutic support, or training, to improve outcomes and reduce harm”. This definition was designed to encompass not only AI applications but also any future digital modality used in the practice of surgery. Despite this broad definition, experts largely focused on AI applications within surgery. Key issues were grouped into six areas: data; privacy, confidentiality, and public trust; consent; law; litigation and liability; and commercial partnerships. Of these, issues of privacy and public trust, consent, and litigation were the most specific to digital surgery.

In terms of privacy, data confidentiality and secure data storage are high priorities—most obviously to protect patient health information, but also to ensure surgeons’ right to privacy. Panelists agreed that public trust has been hampered by the opacity of “black box” decision-making^5^, lack of existing effective surgical AI systems to date, and fear of producing or worsening bias in datasets. AI systems that rely on machine learning are often referred to as “black box” models that cannot be easily interpretable by humans^4^. While patient education about AI models is one option to build public trust, a more accessible and feasible option is mandatory reporting of outcomes when digital technology is used in surgery. On the other hand, reporting outcomes could affect surgeon behavior unless surgeon privacy is maintained.

Panelists also reached a consensus on the need for a standardized methodology for consenting patients to share their data for digital surgery applications. The consent procedure should specify the extent of data collection, who will have access, why the data is being collected, and data management protocols for subsequent applications. Then patients should be asked to consent separately when allowing commercial partners to access their data. Within a surgical context, however, there are once again unique challenges that arise. Collecting data for unspecified future applications is ethically questionable. In addition, the right to withdraw consent is problematic within digital surgery. If an AI system is trained using a particular patient’s data, then a patient cannot realistically withdraw consent or take back their data.

Finally, panelists agreed upon litigation and liability as a pivotal issue of importance. The application of digital technology to data-rich surgical videos has the potential to be key to improvements in the field. However, digital surgical systems may also contribute evidence to determine medical negligence, limiting widespread adoption of otherwise useful digital surgery systems. Currently, no regulatory policies govern liability for either digital surgery systems that fail or surgeons who decide whether or not to use them. A 2018 report by the American Medical Association found that surgeons, along with obstetricians and gynecologists, are already at greatest risk of medical liability lawsuits^6^. To foster continuing innovation, medical, legal, and policy organizations will need to establish standards for legal responsibility with the use of digital surgery. Within this intersection, surgeons with a background in digital technology are well-positioned to lead the charge and develop policy standards in collaboration with non-clinical stakeholders.

The digitization of surgery is here to stay. Along with investments into infrastructure and the development of data-sharing agreements, issues of privacy and public trust, consent, and liability need to be confronted and standardized to capitalize on digital surgery’s potential.

## Data Availability

No datasets were produced or analyzed for this article.

## Code availability

No computer code was produced or analyzed for this article.
